# Effect of active vitamin D3 on VEGF-induced ADAM33 expression and proliferation in human airway smooth muscle cells: implications for asthma treatment

**DOI:** 10.1186/s12931-016-0490-9

**Published:** 2017-01-05

**Authors:** Sung-Ho Kim, Qing-Mei Pei, Ping Jiang, Min Yang, Xue-Jiao Qian, Jiang-Bo Liu

**Affiliations:** 1 Department of Respiration, Tianjin First Central Hospital, Fukanglu-24, Nankaiqu, Tianjin, 300192 China; 2 Department of Radiology, Tianjin Hospital of Integrated Traditional Chinese and Western Medicine, Tianjin, China

**Keywords:** Asthma, Vascular endothelial growth factor, ADAM33, 1,25-dihydroxyvitamin D3

## Abstract

**Background:**

Asthma is a chronic respiratory disease characterized by reversible airway obstruction with persistent airway inflammation and airway remodeling, which is associated with increased airway smooth muscle (ASM) mass. Vascular endothelial growth factor (VEGF) has been implicated in inflammatory and airway blood vessel remodeling in asthma. Recent evidence indicates that a deficiency of 1,25-dihydroxyvitamin D3 (1,25(OH)2D3) may influence asthma pathogenesis. A disintegrin and metalloproteinase (ADAM)33 has been identified as playing a role in the pathophysiology of asthma. ADAM33, which is expressed in ASM cells, is suggested to play a role in the function of these cells. Recent studies show that 1,25-(OH)2D3 exerts direct inhibitory effects on passively sensitized human ASM cells in vitro, including inhibition of ADAM33 expression and cell proliferation; however, the mechanism has not been fully understood.

**Methods:**

In order to elucidate the precise mechanism underlying the effect of 1,25(OH)2D3 on VEGF-induced ADAM33 expression and ASM cell proliferation, we tested the effects of 1,25(OH)2D3 on cell cycle progression and evaluated the levels of phospho-VEGF receptor 2 (VEGFR2), phospho-extracellular signal-regulated kinase 1/2 (ERK1/2), and phospho-Akt in VEGF-stimulated ASM cells.

**Results:**

We found that 1,25(OH)2D3 inhibited VEGF-induced ADAM33 expression and ASM cell proliferation, as well as cell cycle arrest. Additionally, VEGF-induced ADAM33 expression and ASM cell proliferation was suppressed *via* inhibition of ERK1/2 activity, but not that of Akt. Furthermore, 1,25(OH)2D3 treatment inhibited VEGF-induced activation of VEGFR2 as well as that of ERK and Akt in a concentration-dependent manner. 1,25(OH)2D3 also inhibited transforming growth factor (TGF)-β-induced VEGF secretion by ASM cells.

**Conclusions:**

Collectively, our findings suggest that 1,25(OH)2D3 inhibits VEGF-induced ASM cell proliferation by suppressing VEGFR2 and ERK1/2 activation and downregulating ADAM33. Further studies of these mechanisms are needed to facilitate the development of treatments for smooth muscle hyperplasia-associated diseases of the airway such as asthma.

## Background

Asthma is a chronic respiratory disease characterized by reversible airway obstruction with persistent airway inflammation and airway remodeling. Airway remodeling and airway obstruction have several features in common such as airway smooth muscle (ASM) cell hyperplasia and hypertrophy, as well as increase in vascular permeability and angiogenesis [1, 2], both of which have been the target for numerous therapeutic regimens.

Recently, several growth factors and cytokines secreted by inflammatory cells have been implicated in ASM cell growth and division. Among these, Vascular endothelial growth factor (VEGF)-A (hereafter called VEGF) has been implicated in asthma-related inflammation and remodeling [3, 4].

Various cytokines, cellular elements, oxidative stress, and protease/antiprotease levels affect lung fibroproliferation, remodeling, and function, which may be influenced by vitamin D levels [5]. Moreover, previous studies have suggested the active involvement of VEGF in the pathogenesis of asthma, which may be mediated by 1,25-dihydroxycholecalciferol [1,25(OH)2 D3], the active form of vitamin D. In addition, 1,25(OH)2D3 has also been shown to inhibit the proliferation of airway smooth muscle cells [6]. A disintegrin and metalloproteinase (ADAM)33, a recently discovered ADAM family member, has been found to play a role in the pathophysiology of asthma [7]. Similar to other protease-type ADAM members, the active site sequence of ADAM33 lies in the metalloprotease domain, implying that this protein promotes the processing of growth factors, various adhesion molecules, cytokines, and cytokine receptors [8]. ADAM33 is preferentially expressed in smooth muscle cells, fibroblasts, and myofibroblasts, but not in T cells, epithelial cells, or inflammatory cells [9]. ADAM33 has been linked to allergic airway inflammation; however, its role in the pathophysiology of asthma remains to be proven. Recent studies show that 1,25-(OH)2D3 exerts direct inhibitory effects on passively sensitized HASMCs in vitro, including inhibition of cell proliferation and expression of ADAM33 [10, 11]. However, the mechanisms underlying 1,25-(OH)2D3-induced inhibitory effects on ASM cell proliferation and expression of ADAM33 remain poorly defined.

Therefore, in the present study, we aimed to investigate the mechanisms underlying the inhibitory effects of 1,25-(OH)2D3 on VEGF-induced ADAM33 expression and ASM cell proliferation. Evaluation of cell proliferation by bromodeoxyuridine (BrdU) labeling has been described in several cell types and species. The key principle of this method is that BrdU is incorporated as a thymidine analog into nuclear DNA, thus acting as a marker that can be tracked with antibodies [12].

## Methods

### Antibodies and reagents

Antibodies against phospho-ERK1/2 (Thr^202^/Tyr^204^), phospho-Akt (Ser^473^), phospho-VEGFR2 (Tyr^1175^), ERK1/2, Akt, and VEGFR2 were purchased from cell signaling technology (Danvers, MA). Antibody against ADAM33 and β-actin was obtained from Santa Cruz Biotechnology (Santa Cruz, CA). The secondary antibodies were obtained from (Jackson Immunoresearch, West Grove, PA). 1,25-(OH)2D3, TGF-β1, and VEGF were purchased from Sigma-Aldrich (St. Louis, MO). SU1498 was from CalBiochem (La Jolla, CA). PI3-K inhibitor LY294002, and the MAPK/ERK1/2 inhibitor U0126 were obtained from CalBiochem (La Jolla, CA).

### Cell culture

Human ASM cells were obtained from ScienCell Research laboratories. Cells were cultured in six-well plates in Smooth Muscle Cell Medium (SMCM) containing 10% FBS and were maintained at 37 °C and 5% CO_2_ as previously described [13]. Cells from passage 3–6 maintained their SMC phenotype and were used in all experiments. ASM cells were characterized by smooth muscle cell markers including smooth muscle α-actin and smooth muscle heavy chain using immunofluorescence. In inhibition experiments, inhibitors of signal transduction pathways were added for 2 h before the addition of VEGF. All inhibitors were dissolved in dimethyl sulfoxide (DMSO; final concentration of 0.1%, vol/vol) and added to the medium. Vehicle controls contained the same amount of DMSO.

### Real-time reverse transcriptase–PCR

Total RNA was isolated from ASM cells using a TRIzol regent (Invitrogen) after exposure to VEGF or 1,25-(OH)2D3. Total RNA (2 μg) was reverse transcribed using the oligo (dT) primer and MMLV reverse transcriptase (Promega, Madison, WI) at 42 °C for 90 min. Real-time PCR was performed using an ABI Prism 7500 instrument according to the manufacturer’s instructions (Applied Biosystems, Foster City, CA). The following primer pairs were used: ADAM33, forward 5’- CAGGAATGCCAGCTATTATC −3’ and reverse, 5’-GTTTGGTGTGGTTCAAGTTT-3’; and GAPDH, forward 5’-GGCCAAAAGGGTCATCA TC −3’ and reverse, 5’-GTGATGGCATGGACTGTGG-3’. After an initial hot start for 10 min, amplification was performed for 40 cycles consisting of denaturation for 10 s at 94 °C, annealing for 30 s at 56 °C, and extension for 40 s at 72 °C. The amplification kinetics was recorded as sigmoid progress curves for which fluorescence was plotted against the number of amplification cycles. The threshold cycle number (CT) was used to define the initial amount of each template. The CT was the first cycle for which a detectable fluorescent signal was observed. The mRNA expression levels were determined and compared with the GAPDH standard.

### BrdU incorporation assay

ASM cells were seeded in 96-well plates and treated with various drugs as indicated in each experiment for indicated times. At the end of treatment, BrdU incorporation was assayed by incubating the cells with BrdU for 0.5–1 h using a BrdU Cell Proliferation Assay Kit (Calbiochem, San Diego, CA) according to the manufacturer’s instructions.

### Establishment of ADAM33 overexpressing cell lines

To generate ADAM33 overexpressing vectors, the ADAM33-coding sequences were obtained by reverse transcription PCR and cloned into pMXs-based retroviral plasmid (Addgene). Human ASM cells were infected as described [14], to establish ADAM33 overexpressing ASM cells (ASM cells-ADAM33), and ASM cells infected with retrovirus containing blank pMXs vector (ASM cells-vector) were used as the control group.

### Cell cycle analysis

ASM cells were cultured in the complete medium with 1,25-(OH)2D3 for 2 h before treated or not with 50 ng/ml of VEGF for 48 h. All the cells were collected, and 1 × 10^6^ cells were centrifuged, resuspended in ice-cold 70% ethanol and stored at −20 °C until further analysis. Washed cells were stained by 0.1% Triton X-100 in 0.01 M phosphate-buffered saline (pH 7.2) with 50 μg/ml propidium iodide (Sigma-Aldrich) and 1 mg/ml RNase A (Invitrogen), and incubated at 37 °C for 30 min in the dark. Samples of the cells were then analyzed for their DNA content using FACScan flow cytometry (Beckman, Miami, FL), and cell cycle phase distributions were analyzed by the Cell Quest acquisition software (BD Biosciences, Franklin Lanes, NJ). All experiments were performed in duplicate and repeated twice.

### Western blot analysis

The cell extracts were separated by 10% sodium dodecyl sulphate-polyacrylamide gel electrophoresis (SDS-PAGE) and transferred onto a nitrocellulose membrane. The membranes were blocked in blocking solution [5% non-fat dried milk in phosphate buffered saline (PBS)] for 2 h at room temperature and then probed with anti-ADAM33, anti-VEGFR2, anti-P-VEGFR2, anti-P-ERK1/2, anti-ERK1/2, anti-P-Akt, anti-Akt, and anti- β-actin for 1 h at room temperature. After washing three times in phosphate-buffered saline (PBS) containing 0 · 1% Tween-20 (PBS-T), the membranes were incubated with secondary antibodies for 1 h at room temperature. After washing an additional three times in PBS-T, the membranes were developed using an electrochemiluminescence (ECL) solution (Pierce, Rockford, IL, USA) and exposed to Kodak X-ray film.

### Transfection of small interfering RNA (siRNA)

ADAM33 siRNAs were purchased from Santa Cruz Biotechnology (Santa Cruz, CA). ADAM33 was transfected into ASM cells, ASM cells-ADAM33, and ASM cells-vector according to a siRNA transfection protocol provided by Santa Cruz Biotechnology. Briefly, after culturing ASM cells in antibiotic-free SMCM at 37 °C in a humidified atmosphere of 5% CO_2_ for 24 h, the siRNA duplex solution, which was diluted in siRNA transfection medium (Santa Cruz Biotechnology), was added to the ASM cells. After transfection for 24 h, the medium was replaced with normal SMCM, and ASM cells were treated with VEGF. Scrambled siRNA, purchased from Santa Cruz Biotechnology, was transfected to ASM cells as a negative standard.

### Measurement of VEGF secretion

ASM cells were incubated with indicated doses of 1,25-(OH)2D3 for 48 h after stimulation with TGF-β1 for 30 min, and then the VEGF concentration in each supernatant was quantified using an ELISA kit for human VEGF (Invitrogen, Camarillo, CA) according to the manufacturer’s instructions.

### Statistical analysis

All results are expressed as the mean ± SEM. The statistical evaluation of the results was performed by an independent *t*-test and an ANOVA with a Tukey post-hoc test. The results were significant with a value of *p* < 0.05.

## Results

### 1,25-(OH)2D3 inhibits VEGF-induced ADAM33 expression in ASM cells

**Fig. 1:**
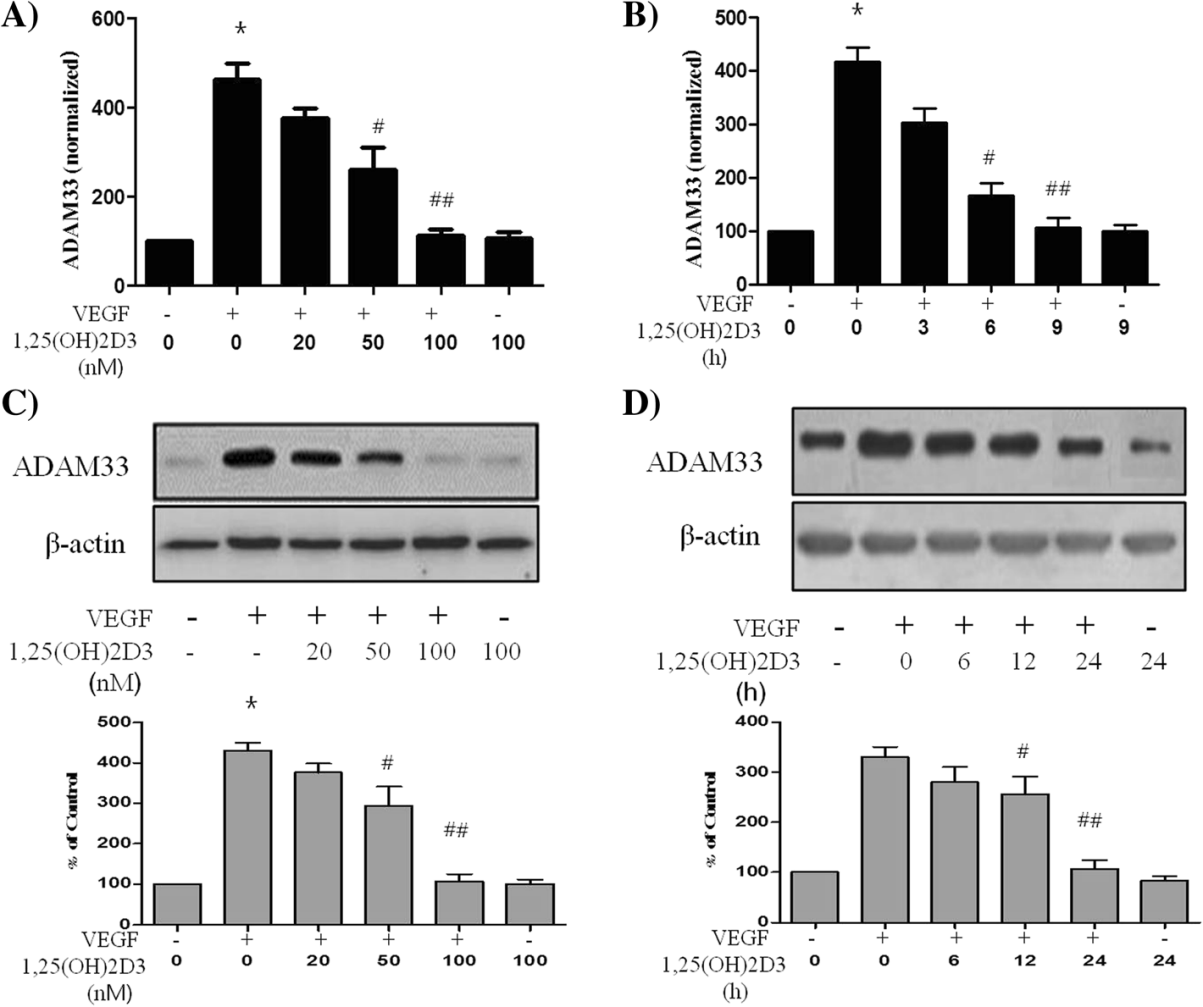
1,25(OH)2D3 inhibits VEGF-induced ADAM33 expression at both mRNA and protein level. ASM cells were incubated with various doses of 1,25(OH)2D3 for 9 h before treatment or not with 50 ng/ml of VEGF for 30 min, and then real-time PCR performed (**a**). ASM cells were incubated at indicated times of 100 nM of 1,25(OH)2D3, and then real-time PCR performed (**b**). The values are normalized relative to the GAPDH standard. ASM cells were incubated with various doses of 1,25(OH)2D3 for 24 h before treatment or not with 50 ng/ml of VEGF for 30 min, and then western blotting analysis for ADAM33 was performed (**c**). ASM cells were incubated at indicated times of 100 nM of 1,25(OH)2D3 before treatment or not with 50 ng/ml of VEGF for 30 min, and then western blotting analysis for ADAM33 was performed (**d**). β-actin was used as a loading control. All data are representative of three independent experiments. Values represent the means  ±  SEM. ^*^ *P*  <  0.05 *vs.* control, ^#^ *P*  <  0.05, ^##^ *P*  <  0.005 *vs.* VEGF alone

In order to evaluate the direct effect of 1,25-(OH)2D3 on the VEGF-induced expression of ADAM33 in ASM cells, we performed real-time PCR. ASM cells were treated with various doses of 1,25-(OH)2D3, and at various times after treatment or not with 50 ng/ml of VEGF for 30 min; as a result, VEGF-induced ADAM33 expression was downregulated in a dose- and time-dependent manner (Fig. 1a, b). In addition, we performed western blot analysis to evaluate whether 1,25-(OH)2D3 additionally regulates VEGF-induced ADAM33 protein expression in ASM cells. Interestingly, 1,25-(OH)2D3 also downregulated VEGF-induced ADAM33 protein expression in ASM cells in a dose- and time-dependent manner (Fig. 1c, d).We did not observe any VEGF- and/or 1,25-(OH)2D3-induced cytotoxicity under the present experimental conditions (data not shown).

### 1,25-(OH)2D3 inhibits VEGF-induced ASM cell proliferation by downregulating ADAM33 expression

**Fig. 2:**
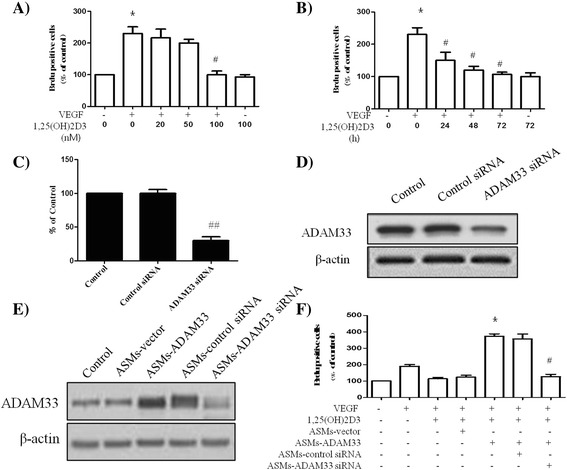
1,25(OH)2D3 inhibits cell proliferation by down-regulation of ADAM33 expression. ASM cells were incubated with various doses of 1,25(OH)2D3 for 48 h before treatment or not with 50 ng/ml of VEGF for 30 min, and then cell proliferation was determined by BrdU incorporation (**a**). ASM cells were incubated at indicated times of 100 nM of 1,25(OH)2D3 before treatment or not with 50 ng/ml of VEGF for 30 min, and then cell proliferation was determined by BrdU incorporation (**b**). ASM cells were transfected with negative siRNA or ADAM33 siRNA, and then real-time PCR performed. The values are normalized relative to the GAPDH standard (**c**). ASM cells (**d**) and ASM cells-ADAM33 (**e**) were transfected with negative siRNA or ADAM33 siRNA, and then western blotting analysis for ADAM33 was performed. β-actin was used as a loading control. ASM cells-ADAM33 were transfected with negative siRNA or ADAM33 siRNA in the presence of VEGF (50 ng/ml) and 1,25-(OH)2D3 (100 nM) for 48 h, and then cell proliferation was determined by BrdU incorporation (**f**). All experiments were done at least three times. Values represent the means  ±  SEM. ^*^ *P*  <  0.05 *vs.* control or ASMs-vector; ^#^ *P*  <  0.05 *vs.* VEGF alone or control siRNA or ASMs-control siRNA

It has been reported that VEGF-D-enhanced ADAM33 plays an important role in tumor cell proliferation in the gastric cancer cell line SNU-601. We initially tested the effect of 1,25-(OH)2D3 on the VEGF-induced proliferation of ASM cells. When ASM cells were treated with various doses of 1,25-(OH)2D3, and at different times after treatment or not with 50 ng/ml of VEGF for 30 min, 1,25-(OH)2D3 inhibited VEGF-enhanced BrdU incorporation in a dose- and time-dependent manner in ASM cells (Fig. 2a, b).

Next, to elucidate the effect of ADAM33 on the proliferation of ASM cells, we constructed an ADAM33 siRNA transfection reagent. As shown in Fig. 2c and d, we confirmed ADAM33 gene silencing at the mRNA and protein level. To further confirm the silencing effect of ADAM33 siRNA in ASM cells, a rescue experiment was performed with ADAM33 siRNA in ASM cells-ADAM33. Herein, western blot analysis was also performed to assess ADAM33 expression in ASM cells-ADAM33 treated with ADAM33 siRNA (ASM-ADAM33 siRNA). The result of western blot analysis indicated that the expression of ADAM33 was significantly downregulated in ASM-ADAM33 siRNA compared with ASM cells-ADAM33 and ASM cells-ADAM33 treated with nontargeting control siRNA (ASM-control siRNA) (Fig. 2e). These results indicated that the ADAM33 siRNA was effective in our study.

The cell proliferation ability was further evaluated. As expected, When ASM cells-ADAM33 cells were transfected with ADAM33 siRNA or control siRNA for 48 h in the presence of 50 ng/ml VEGF and 100 nM 1,25-(OH)2D3, BrdU incorporation was decreased in ADAM33 siRNA-transfected cells compared with negative control siRNA-transfected cells (Fig. 2f). These data indicate that 1,25-(OH)2D3 inhibits VEGF-induced proliferation of ASM cells by downregulating ADAM33 expression.

### 1,25-(OH)2D3 induces G1-phase cell-cycle arrest in VEGF-induced ASM cell proliferation

**Fig. 3:**
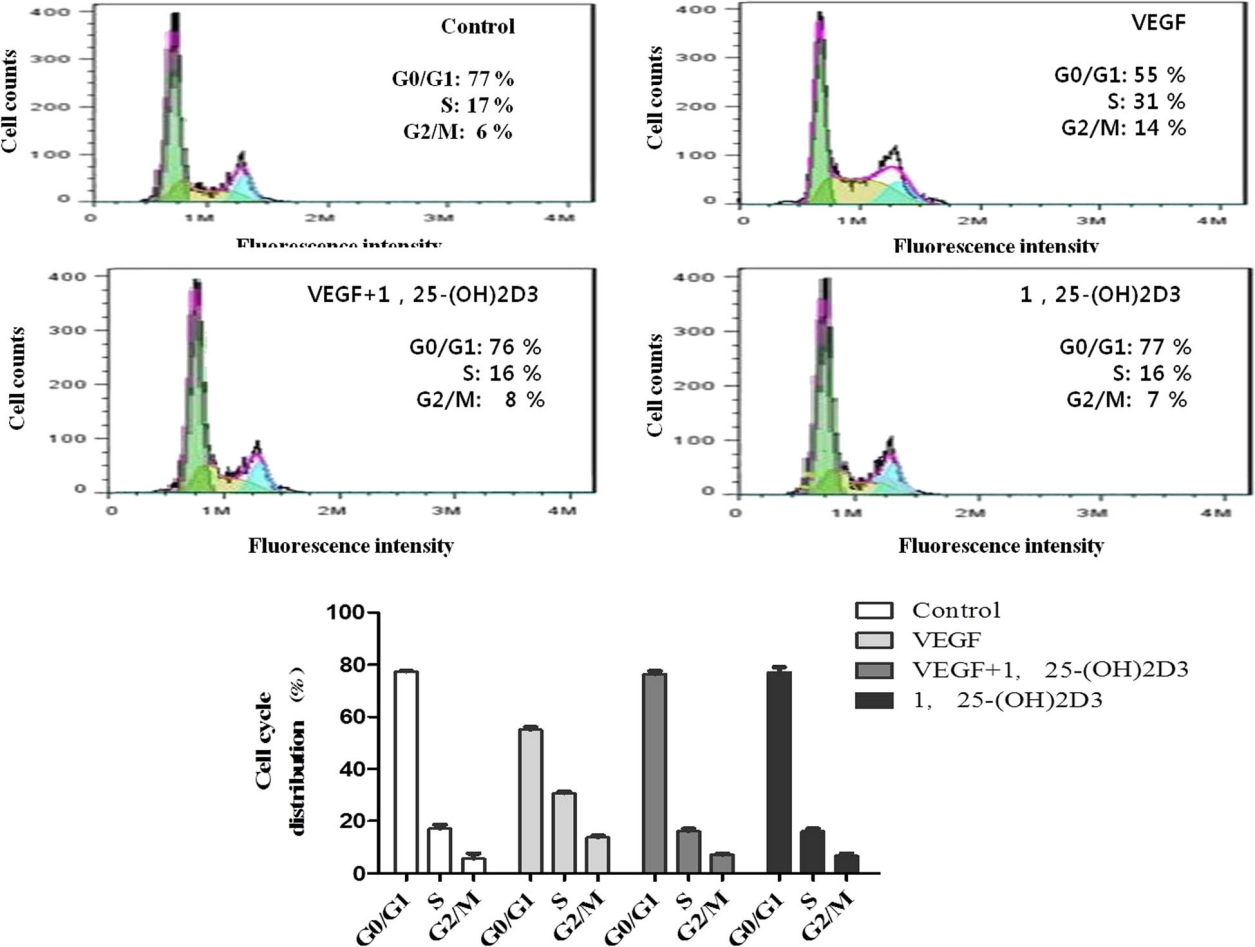
1,25(OH)2D3 inhibits cell cycle in ASM cells. ASM cells were incubated with 100 nM of 1,25(OH)2D3 in the presence or absence of VEGF (50 ng/ml) for 48 h, and then Flow cytometric analysis for cell cycle was performed. All experiments were done at least three times. Values represent the means  ±  SEM

Flow cytometry analysis was performed to assess whether the anti-proliferative effect of 1,25-(OH)2D3 was due to cell-cycle arrest in a specific phase. As shown in Fig. 3, VEGF treatment significantly increased the proportion of ASM cells in the S and G2/M phases of the cell cycle, with a concomitant decrease in the proportion in G1 phase relative to control cells. However, 1,25-(OH)2D3 treatment in the presence or absence of VEGF markedly reduced the percentage of cells in the S and G2/M phases, resulting in a significant accumulation of cells in G1 phase, relative to VEGF-stimulated ASM cells. Cell proliferation was only slightly affected by 1,25-(OH)2D3 alone compared with the group without VEGF challenge.

### 1,25-(OH)2D3 inhibits VEGF-induced ADAM33 expression and ASM cell proliferation through inactivation of VEGFR2 (KDR/Flk1)

**Fig. 4:**
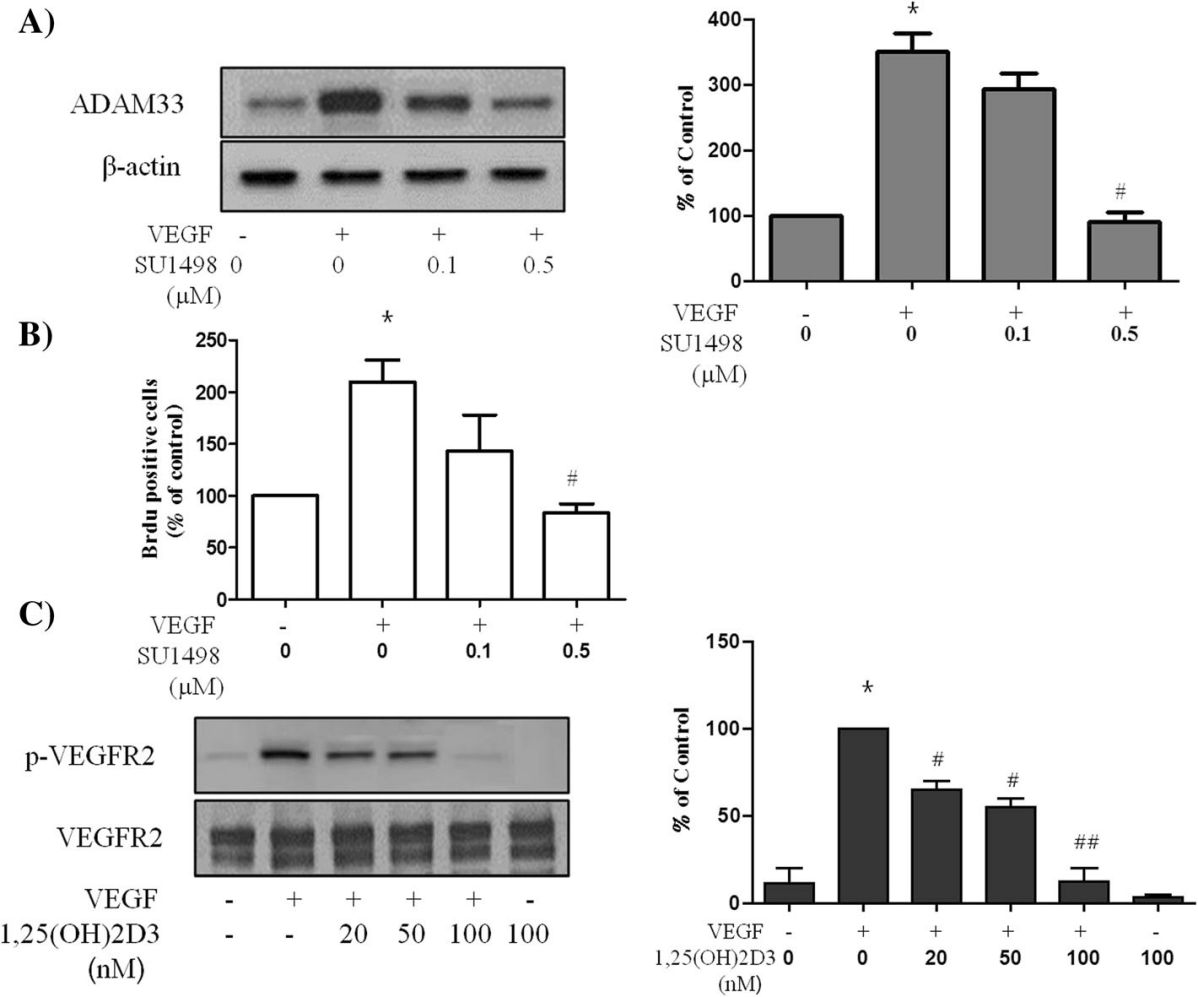
1,25(OH)2D3 inhibits VEGF-induced ADAM33 expression and cell proliferation by inactivation of VEGFR2. ASM cells were incubated with indicated doses of SU1498 for 2 h before treatment with VEGF (50 ng/ml) for 24 h, and then western blotting analysis for ADAM33 was performed. β-actin was used as a loading control (**a**). ASM cells were incubated with indicated doses of SU1498 for 2 h before treatment with VEGF (50 ng/ml) for 48 h, and then cell proliferation was determined by BrdU incorporation (**b**). ASM cells were incubated with various doses of 1,25(OH)2D3 for 24 h before treatment or not with 50 ng/ml of VEGF for 30 min, and then western blotting analysis for VEGFR2 was performed (**c**). All experiments were done at least three times. Values represent the means  ±  SEM. ^*^ *P*  <  0.05 *vs.* control; ^#^ *P*  <  0.05, ^##^ *P*  <  0.005 *vs.* VEGF alone

Several studies have reported that VEGF-induced cell proliferation is mediated by the interaction of VEGF with VEGFR2 (also known as KDR or FLK1). In order to determine whether blocking the VEGF-VEGFR2 interaction prevents VEGF-mediated ADAM33 upregulation and ASM cell proliferation, we used SU1498, an inhibitor of the tyrosine kinase activity of VEGFR2. SU1498 blocks the interaction of VEGF with VEGFR2 but not with VEGFR1 (FLT1). As shown in Fig. 4a, VEGF-increased ADAM33 expression was inhibited by SU1498 in a dose-dependent manner. In addition, SU1498 blocked VEGF-induced BrdU incorporation in ASM cells (Fig. 4b). Finally, we performed western blot analysis to evaluate whether 1,25-(OH)2D3 also regulates VEGF-induced VEGFR2 activation in ASM cells. Interestingly, 1,25-(OH)2D3 also downregulated VEGF-induced VEGFR2 activation in ASM cells in a dose-dependent manner. These data indicate that 1,25-(OH)2D3 inhibits VEGF-induced ADAM33 expression and cell proliferation by suppression of the VEGF/VEGFR2 interaction.

### 1,25-(OH)2D3-mediated inhibition of VEGF-induced ADAM33 expression and cellular proliferation involves the MAPK/ERK1/2 pathway

**Fig. 5:**
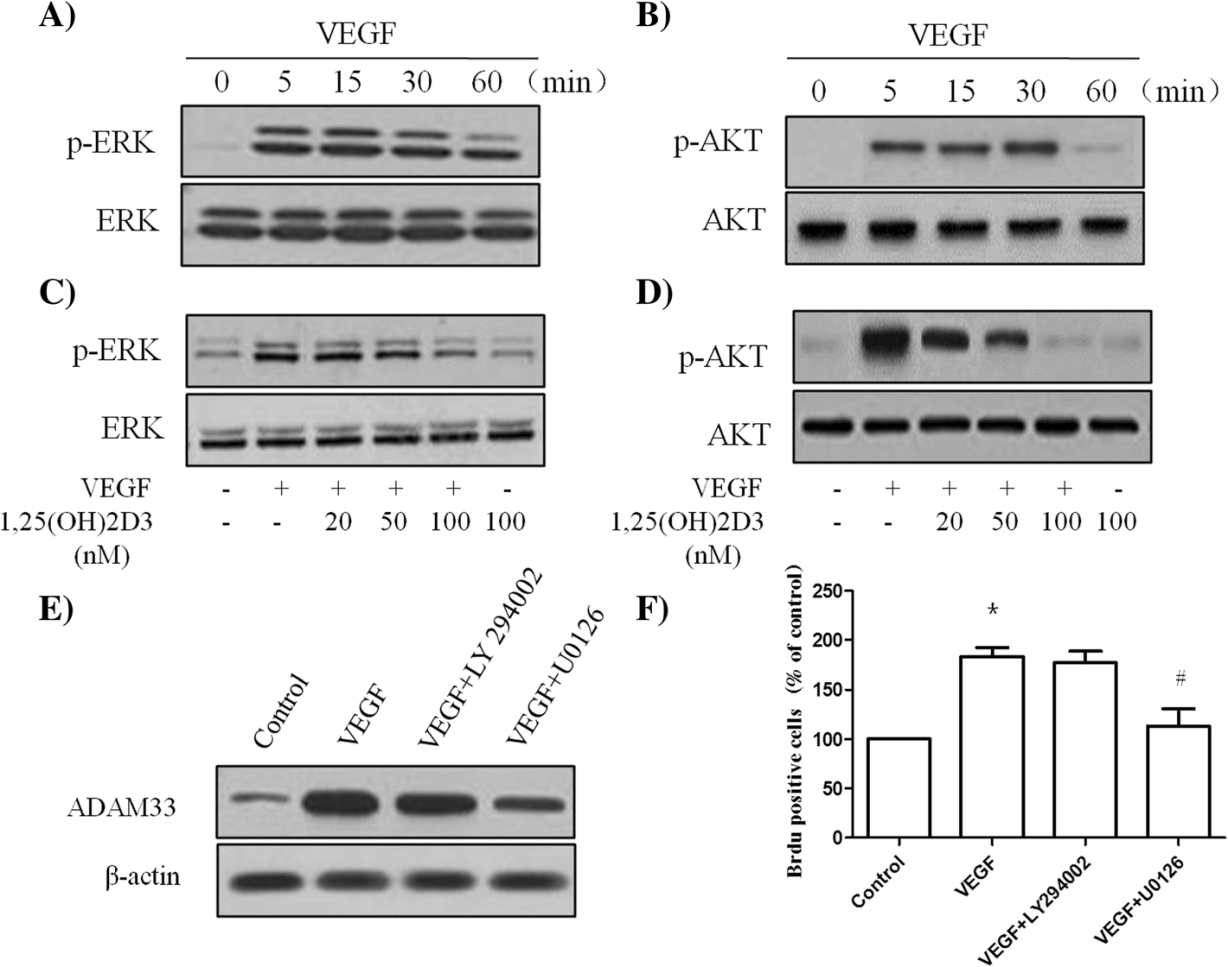
1,25(OH)2D3 inhibits VEGF-induced ERK 1/2 phosphorylation in ASM cells. ASM cells were incubated at indicated times of VEGF (50 ng/ml), and then western blotting analysis for phospho-ERK 1/2 (**a**) and phospho-Akt (**b**) was performed. ASM cells were incubated with various doses of 1,25(OH)2D3 for 24 h before treatment or not with 50 ng/ml of VEGF for 30 min, and then western blotting analysis for phospho-ERK 1/2 (**c**) and phospho-Akt (**d**) was performed. The total ERK1/2 and Akt was used as a loading control. ASM cells were incubated with 20 μM U0126 or 20 μM LY294002 for 2 h before treatment with VEGF (50 ng/ml) for 24 h, and then western blotting analysis for ADAM33 was performed. β-actin was used as a loading control (**e**). ASM cells were incubated with 20 μM U0126 or 20 μM LY294002 for 2 h before treatment with VEGF (50 ng/ml) for 48 h, and then cell proliferation was determined by BrdU incorporation (**f**). All experiments were done at least three times. Values represent the means  ±  SEM. ^*^ *P*  <  0.05 *vs.* control; ^#^ *P*  <  0.05 *vs.* VEGF alone

The majority of in vitro reports suggest that PI3-K and ERK1/2 activation represents the major signal transduction pathway for cytokine-stimulated, G-protein-coupled-receptor (GPCR)-mediated, or receptor tyrosine kinase (RTK)-mediated proliferation of ASM cells. It is known that the interaction of VEGF with VEGFR2 activates the MAPK/ERK1/2-dependent and PI3-k/Akt-dependent signaling transduction pathways. In order to evaluate the effects of VEGF and 1,25-(OH)2D3 on the activation of ERK1/2 and Akt in ASM cells, the levels of phosphorylated ERK1/2 and Akt expression were investigated by western blotting. When cells were incubated in the absence or presence of 50 ng/ml VEGF for the indicated duration, an increase in the phosphorylation of ERK1/2 and Akt was observed, with no effect on the total levels of these proteins (Fig. 5a and b). In addition, treatment with 1,25-(OH)2D3 significantly attenuated VEGF-stimulated ERK1/2 and Akt phosphorylation in a dose-dependent manner (Fig. 4c and d). The levels of phospho-ERK1/2 and phospho-Akt were not affected in 1,25-(OH)2D3-treated cells compared with the group without VEGF challenge.

In order to determine the signaling pathways that play a role in VEGF-induced ADAM33 expression and ASM cell proliferation, we used specific inhibitors of ERK1/2 and Akt signaling pathways. The inhibitors tested included U0126 (a MAPK/ERK1/2 inhibitor) and LY294002 (a PI3K inhibitor). Pretreatment of ASM cells with U0126 was found to significantly decrease ADAM33 expression in VEGF-treated ASM cells relative to control (Fig. 5e). However, VEGF-induced ADAM33 expression was not affected by the addition of LY294002 (Fig. 5e). These results demonstrate that VEGF increases ADAM33 expression through the activation of ERK1/2. We additionally investigated whether U0126 and LY294002 inhibit the VEGF-induced proliferation of ASM cells; when cells were incubated with U0126 or LY294002in the presence of VEGF, U0126 blocked VEGF-induced BrdU incorporation (Fig. 5f). However, LY294002 had no effect on VEGF-induced BrdU incorporation (Fig. 5f). These data show that 1,25-(OH)2D3 inhibits VEGF-induced ADAM33 expression and proliferation of ASM cells through the suppression of the MAPK/ERK1/2 pathway.

### Effect of 1,25-(OH)2D3 on VEGF secretion in ASM cells

**Fig. 6:**
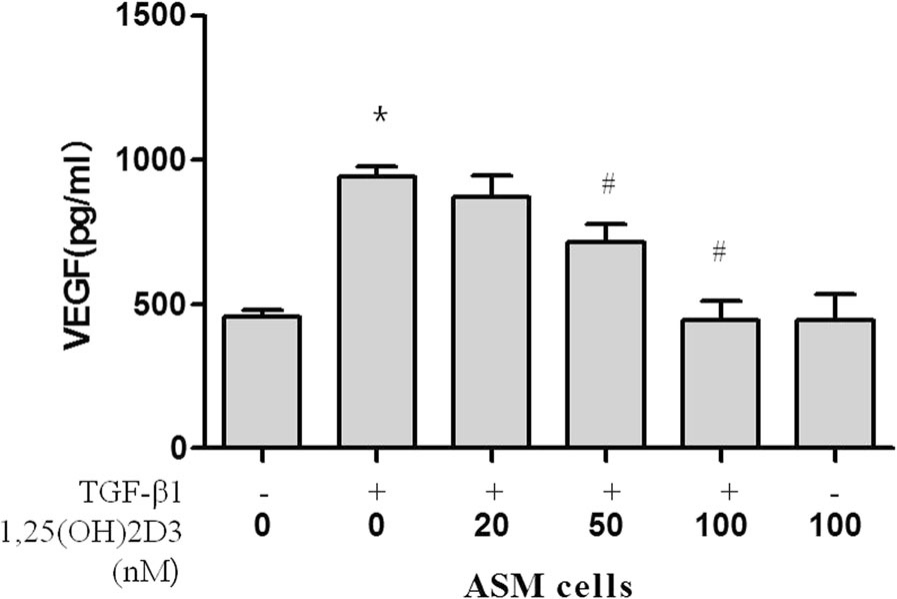
1,25(OH)2D3 inhibits TGF-β1-stimulated VEGF secretion by ASM cells. ASM cells were incubated with indicated doses of 1,25(OH)2D3 for 48 h after stimulation with 10 ng/ml of TGF-β1 for 30 min, and then the VEGF concentration in each supernatant was quantified using a human ELISA kit. Experiments were done at least three times. Values represent the means  ±  SEM. ^*^ *P*  <  0.05 *vs.* control; ^#^ *P*  <  0.05 *vs.* TGF-β1 alone

VEGF released from airway epithelial cells aggravates airway inflammation and remodeling. TGF-β1 expression is elevated in asthmatic airways as well as in the bronchoalveolar lavage (BAL) fluid of patients with asthma. Therefore, we studied the effects of 1,25-(OH)2D3 on TGF-β1-induced VEGF expression in ASM cells. The VEGF levels in culture supernatants from TGF-β1-stimulated ASM cells were significantly higher than those in unstimulated cells (Fig. 6). However, 1,25-(OH)2D3 inhibited the release of VEGF from ASM cells stimulated with TGF-β1 in a dose-dependent manner (Fig. 6). TGF-β1 contributes to airway inflammation by enhancing VEGF release *via* the PI3-K pathway in human ASM cells. Therefore, these data suggest that 1,25-(OH)2D3 inhibits TGF-β1-induced VEGF release, likely by attenuating Akt phosphorylation.

## Discussion

Elucidating the regulatory mechanisms of human ASM cell proliferation has potential clinical value, and may provide further insights into new strategies for the treatment of airway diseases, such as asthma, associated with smooth muscle hyperplasia.

VEGF is a potent stimulator of angiogenesis in asthma. Studies have found that epithelial cell-secreted VEGF promotes airway remodeling in asthma [15]. In addition, VEGF levels are elevated in lung tissues and sputum of patients with asthma, and positively correlate with asthma disease severity. Furthermore, inhibition of VEGF leads to a significant reduction in goblet cell hyperplasia and basement membrane thickness [16, 17]. A previous study found that ASM cells under strain promote angiogenesis *via* secretion of VEGF, and that blocking VEGF attenuated these angiogenic changes [18]. In our previous study, we found that basal VEGF release from human ASM cells was comparable to that from BEAS-2B cells, a human lung epithelial cell line. VEGF secreted by ASM cells is thought to play a role in extracellular matrix modulation and fibronectin secretion, as well as in smooth muscle hypertrophy, and therefore in remodeling [19, 20]. Therefore, in the present study, we investigated the effect of 1,25(OH)2D3 on VEGF-induced cell proliferation and relevant signal transduction pathways, as well as on TGF-β1-induced VEGF secretion in human ASM cells.

ADAM33 was investigated as a potentially important molecule in ASM cell proliferation for a number of reasons. ADAM33 has been shown to be expressed in ASM cells and airway fibroblasts, suggesting a role for this gene in modifying cellular functions such as proliferation, migration, and differentiation [7, 9].

Indeed, ADAM33 expression was significantly higher in the airways of human subjects with asthma compared to those of controls. Further, increased expression correlated with asthma severity progression, from mild to severe lung function [21, 22]. As ADAM33 is predominantly expressed in ASM cells, Lin et al. investigated whether ADAM33 protein expression correlates with ASM cell mechanics in an ovalbumin- (OVA-) sensitized rat model; ADAM33 expression was found to be elevated in ASM cells from OVA-sensitized rats relative to non-sensitized rats. Importantly, ADAM33 expression positively correlated with cell traction force, stiffness, and expression of F-actin and vinculin, suggesting that ADAM33 is a mediator of ASM cell dysfunction in asthma [23]. Although the mechanisms for ADAM33-mediated remodeling are not clear, it has been reported that a soluble form of ADAM33 causes rapid induction of neovascularization both ex vivo and in vivo, as well as endothelial cell differentiation in vitro, suggesting that ADAM33 promotes angiogenesis and elicits airway remodeling [24]. Ito et al. investigated ADAM33 expression in ASM cells and found that ADAM33 mRNA and protein levels are significantly higher in these cells from subjects with asthma than in ASM cells from normal control subjects [9]. In this study, we demonstrated that 1,25(OH)2D3 inhibits VEGF-induced ADAM33 expression at both the mRNA and protein level, suggesting that expression may be primarily regulated at the mRNA level; however, it is necessary to study the ADAM33 promoter region as well as the transcription factors that putatively interact with this region in order to elucidate the pathways involved in the regulation of ADAM33 expression.

1,25(OH)2D3 has been shown to inhibit the proliferation of airway smooth muscle cells [6]. Moreover, in utero vitamin D deficiency in mice leads to increased airway smooth muscle mass and airway resistance [25]. Furthermore, in children with severe asthma, lower levels of vitamin D have been shown to be associated with increased airway smooth muscle mass [26, 27]. Recently, 1,25(OH)2D3 has been reported to exert a negative regulatory effect on VEGF secretion [5, 28]. The mechanism by which 1,25(OH)2D3 regulates VEGF secretion is currently unclear. However, several possible underlying mechanisms have been suggested such as the rapid induction of non-transcriptional responses, which may occur *via* activation of transmembrane signal transduction pathways, *e.g.* protein kinase C, phosphatidylinositol 3-kinase/Akt, and p42/p44 MAP kinase, all of which are closely associated with VEGF expression [5, 29]. In addition, it has been shown that 1,25(OH)2D3 induces rapid and sustained activation of phosphatidylinositol 3-kinase/Akt in a nongenomic manner [5]. Swain et al. reported that 1,25(OH)2D3 may regulate phopholipase C production by cells, which, in turn, may modulate signal transduction by receptors with tyrosine kinase activity, including VEGF [5, 30]. Second, 1,25(OH)2D3 may modulate the expression of growth factor receptors [5, 31]. Finally, growth factors may modulate the expression of the nuclear vitamin D receptor [32]. Further studies are needed to elucidate the mechanisms by which 1,25(OH)2D3 decreases VEGF secretion; this information should facilitate the development of new therapeutic strategies for the treatment of asthma.

It has been found that ERK1/2 activation is involved in cell growth, morphogenesis, and migration of endothelial cells stimulated by angiogenic factors [33, 34]. Moreover, the activation of PI3K/Akt is considered to play a role in a variety of biological functions such as cell growth, vascular remodeling, angiogenesis, and survival [34, 35]. VEGF promotes endothelial cell growth and survival *via* the ERK1/2 and PI3K/Akt pathways, respectively [36–38]. Walker et al. [39] compared the extent to which ERK1/2 or PI3K cascades contributed to α-thrombin-stimulated or PDGF-stimulated proliferation of bovine tracheal smooth muscle, and found that, although the PI3K pathway was essential, the ERK1/2 pathway was required for a full mitogenic response. Such findings suggest that although active PI3K is sufficient for the stimulation of ASM DNA synthesis, either by GPCR-coupled or RTK-coupled pathways, parallel ERK1/2-dependent signaling events are required for maximal proliferation. Recent studies have shown that ERK1/2 activation, but not Akt activation, is required for HASM cell proliferation [40–42]. In the present study, we observed that ERK inhibition, but not PI3K inhibition, suppressed ADAM33 expression induced by VEGF. Further research is needed to elucidate the mechanism by which the ERK1/2 pathway enhances the transcription of ADAM33.

## Conclusion

In conclusion, our results provide important insights into the mechanisms by which 1,25(OH)2D3 regulates VEGF-induced ADAM33 expression and ASM cell proliferation, as the effects of this compound on various underlying cellular signaling pathways such as the suppression of VEGFR2 and ERK1/2 phosphorylation. The present findings expand our knowledge of the role of 1,25(OH)2D3 in airway remodeling, and are expected to enable the development of effective therapies for airway diseases such as asthma.

## Abbreviations

1,25(OH)2D3: 1,25-dihydroxyvitamin D3
ADAM-33: A disintegrin and metalloproteinase33
ASM: Airway smooth muscle
ERK1/2: Phospho-extracellular signal-regulated kinase 1/2
GPCR: G-protein-coupled-receptor
RTK: Receptor tyrosine kinase
TGF-β: Transforming growth factor-β
VEGF: Vascular endothelial growth factor
VEGFR2: Vascular endothelial growth factor receptor 2
